# The Coster's Customer: Or, How I Earned Twenty Pounds

**Published:** 1887-02-26

**Authors:** 


### The Coster's Customer; or, How I earned Twenty Pounds.

By an Amateur Butler.

I am a professional greengrocer and amateur butler. Business has been very bad in both my occupations of late. The people in the neighbourhood have ceased to give dinners—on my honour, I have not seen an *entrée* for six months, and I have almost forgotten the taste of champagne—and in the calm of domestic life my customers accompany their simple joints only with the vulgar potato, neglectful of the delicate charm of sea-kale or the graceful dignity of asparagus.

These are sad times; and to a man who has frequently reflected on the fickleness of fortune and the scanty profits in his till, the chance of earning twenty pounds, "in an easy and genteel manner," as the advertisements put it, was not to be slighted. This chance, however, did not come by advertisement. No; I'm too wary to be deluded in that way. I will not send twelve stamps to X.Y.Z., at the post-office, Mudford, to learn that I may become an agent for the "Little Treasure" domestic knife and scissors grinding-machine, after purchasing a specimen grinder for three-and-sixpence; neither will I undertake the sale of Old Mother Hubbard's Tonic Sedative,—"the medicine of the future," the advertisement says; but, unfortunately, not of the present.

The chance of earning the sum of twenty pounds for doing next to nothing was offered me by a customer of my own. He is an elderly, unmarried gentleman, connected with a bank in the City, who has long been in the habit of calling on me on his way home and purchasing a bunch of radishes or watercress, which he carries home himself. I like people who don't want to have all their things sent, but I'm not sure that I respect them. This gentleman—Mr. Johnson his name was—and I had become friends after a fashion. We discussed questions of general interest—the weather, politics, and the like.

"The G.O.M. is going it, ain't he, sir?" I would say; or, "I wonder what Lord Randy'll be up to next;" and he invariably answered, "Ah, Jebb, we never know what will happen."

And that's true enough, as far as Mr. Johnson himself is concerned.

He had been looking worried and depressed for some weeks, and hadn't been as particular about the freshness of his cresses as I had seen him; but I didn't suspect that there was anything wrong with him. One evening, however, he lingered, after he had made his usual investment, and fidgeted about, touching one thing after another that he never meant to buy, till he had worried me considerably. I tried to talk of Mr. Parnell, but he evidently wasn't interested in the Irish question, and suddenly he asked, "Jebb, would you like to earn twenty pounds without much trouble?"

"Why, sir," said I, "who wouldn't?" And indeed I can't imagine anyone so stupid.

"Well, I can put you in the way of it," Mr. Johnson went on. "It isn't exactly a recognised employment that I want you to enter into. It's a thing that's done pretty frequently by amateurs; but there's only one professional hand, and his methods are painfully limited; I should not wish you to adopt them."

"But," I suggested, "if it's not the greengrocering nor the waiting, I've had no training in—whatever it is."

"I know," he answered, "but it's not a thing that requires much training, and I'll do all I can to facilitate your task."

"But what am I to do?" I asked.

He hesitated. "This is rather too public a place to mention the matter in," he said, at last. "Come to my rooms this evening after you shut your shop, and I will explain to you."

I went to the address he gave me, after business hours, and was civilly received by my customer. He gave me a glass of brandy-and-water, saying I should want it to brace my nerves for the task he meant to impose on me; but, bless you, all the brandy in the world wouldn't have made me fit for what he wanted me to do. I made a few inquiries about the mysterious task, but his answers were vague and indirect. He began by telling me something of his history.

"I am a cashier in a City bank," he said. "I have worked my way up by energy and, my employers say, by stainless honesty: that's all they know about it. And yet I am not selfishly dishonest; I would starve rather than steal the money to buy a meal. Yes, Jebb, I am honest as far as I myself am concerned; my worst enemy could never say I robbed for my own benefit."

He grew terribly excited. I listened, frightened and bewildered, but still at a loss to guess whither his thoughts tended.

"But one can't live in London," Mr. Johnson went on, "without seeing sorrow and desolation that could often be averted by just a little money, so little, so very little, that it seemed a sin it could not be forthcoming. I never professed to work among the poor, after the fashion of some men, searching out cases of distress and applying mechanical, stereotyped remedies. I searched for nothing, but I could not help hearing of individuals going under in this vast river of life for lack of a hand to help them to the shore; of families threatened with starvation or sin, because they could not hang out for a year or two longer—sometimes only a month or two—after which they could make way for themselves. I have sinned for others, many of whom were neither kin nor kin to me; nothing but fellow mortals whom I could save, but save by sin alone."

He rose to his feet, he held his arms aloft in the air, and I saw on his face an expression I had never before beheld in mortal man; but which reminded me somehow of some of those old pictures of saints and the like which I had seen in the National Gallery. I was there once on a Bank Holiday.

"I'll not say that right is wrong," he exclaimed; "that theft is charity, or honour falsehood more than truth; but though God may—will—condemn me eternally for what I have done, I cannot wholly repent of it. I have seen the colour come back to pale faces and the light to weary eyes; I have known hearts beat in more contented unison with the laws of heaven and earth, through my sin—my sin, which is none the less criminal."

"But," I cried, nigh out of my wits at all this, "I can't make you out, sir. What sin, more than what we're all liable to, and is scored off in church on Sunday, have you ever committed that you should be so troubled? As for honesty, you're as honest as the day, and never wronged me of a penn'orth of green-stuff."

"You! why should I wrong you? But I've wronged my employers. I've been at it for years, taking a little at a time and making false entries to cover the theft. They haven't found me out—they believe in me yet; but it can't go on much longer, for the defalcations amount to five thousand pounds."

"Five thousand pounds!" I repeated, aghast.

"Hush! don't speak of it; they need never know; they shall not while I live, and what does it matter afterwards?"

"But how can you hide it, sir? You can't replace such a sum as that, and, sooner or later, there's bound to be an investigation, and then—— Lor, sir, whatever did you do it for?"

"Never mind about that. I can replace it—at the cost of my life."

"That's a heavy price, though."

"I don't think so. I might spend the rest of my days in penal servitude, a shame to myself and a burden to others, without undoing the consequences of my crime; but by dying I can make all straight once more. Listen; I have insured my life for five thousand pounds, the very sum I have taken from the bank, to which I have bequeathed it in my will. My intention when I did so was to take a dose of laudanum—chloral—any of those poisonous drugs which can be bought as sedatives without exciting much inquiry; but I find that suicides forfeit the benefits of their policy. If it were known or suspected that I had taken my own life, the insurance office would refuse to pay the money, and all would be a failure. You see my dilemma. I want to die, but I must not do so by my own hand. You can help me. I offer you twenty pounds to kill me."

"But—but—good heavens!" I gasped, "I don't want to kill you."

"Of course not," he answered, as calm as if he spoke of taking his dinner instead of his life. "The trouble is that nobody wants to kill me, or I might get my job done at a cheaper rate. But to oblige me—and to get twenty pounds—"

"Not for double the money, not for a thousand pounds!" I cried. "What do you mean, sir? It would be murder. I can't do it. Me a greengrocer, too; not even a butcher, who is used to killing!"

"My good Jebb, all killing isn't murder, or where would the soldiers and the hangman be? You may call this a judicial execution if you like."

"That's all very well, but I guess as how the police wouldn't call it that when they found it out. I have the strongest objections to killing you, Mr. Johnson, for your own sake; but even if I could get over these, I must refuse to do it for mine. You may want to die in a sudden and inconvenient fashion, but you don't suppose *I* want to be hanged, do you?"

"Ah! I see," he said, thoughtfully; "your anxiety is natural. I selected you from out all the people I knew, because you were a solitary man like myself; and if by chance any inconvenient consequences should arise from your obliging me, your death would cause no sorrow to anyone. No one would miss you, Jebb."

"That's as you think, sir. I fancy my customers might feel my loss. Though I says it as shouldn't, it isn't everybody that's as punctual and obliging as I am, nor there ain't many who can tell a foreign potato from a home-grown one at a glance. And even if nobody else missed me—why! I should miss myself."

I had to argue it out with him, for I saw now that he was as mad as a hatter, and I was afraid to contradict him too flatly, lest he should take it into his head to sacrifice me as well as himself.

"I suppose there is something in what you say," he answered; "at least I am willing to make allowance for your not being so indifferent to life as I am. But there's very little danger of your act being discovered. My landlady has gone out: I took care of that. We are alone in the house. Everything is as simple as can be: the only thing left to decide is what means you ought to employ."

"I decline to employ any means. You're not a very great customer, but you're regular; and my neck's my neck—the only one I've got."

He took no notice of what I said; he didn't seem to have heard me, and went on discussing the point his mind was fixed on.

"Poison, of course, would be the pleasantest death for me, and the least likely to arouse suspicion before you were out of the way. It would also be the easiest for you to manage; but it has its disadvantages. Some one would be sure to suggest that I had taken it myself, and the insurance company would jump at the idea of suicide to avoid paying the money. I have a revolver here——"

"I can't shoot," I interposed. "I've got no eye, and no notion of aiming. I never could even hit a sparrow with a stone."

"A very poor shot could do what I want," replied Mr. Johnson, speaking quite mildly, as if he were arguing with a fractious child. "If you put the pistol against my head—say at the ear—you couldn't possibly miss me. Still, the report might be heard by the neighbours, and would excite suspicion. You might cut my throat, unless you have a nervous fear of the sight of blood."

"I loathe the thought of it," I cried. "I can't even touch hare-soup, which some folks think delicious."

"That's a great pity—a great pity," he said; and whether he was thinking of my not liking the soup, or not liking to murder him, I can't say.

I was frightened, so horribly frightened that my brain lost the confusion it had previously been in, and I became calm enough to see a way out of the difficulty.

"You put it so clearly," I said, "and you want to leave the world from such good motives, that I really feel I can't disoblige you, though I must say it's as painful a business to me as it can be to you—at least nearly. But there's one troublesome point. I told my errand-boy of the kind invitation you had given me, and you know it would look rather queer if it transpired that you were found dead immediately after I left you. I can overcome my unwillingness to kill you, in consideration of various things; but I can't bring myself to look kindly on being hanged for doing so. That's a point I can't reach by a long way. But I'll tell you what I'll do: I'll burgle you."

"You'll what?"

"If you will take care that the door is left open, or if you will give me your latch-key, I'll enter the house at the midnight hour,"—I tried to make my description as effective as I could—"and then, being for the time your hereditary enemy, who hates you for being better than himself, I'll rob and murder you. I don't want your watch or other valuables—the possession of them might arouse suspicion—but I shall be obliged if you will put them out of sight—pawn them, if you like—so that it may seem that the mysterious midnight thief has taken them. Perhaps you'll be as kind also as to make your room as untidy as possible—overturn the furniture, and tumble the contents of your bureau on the floor, that it may look as if I had been searching for further treasure. Then I'll tie your hands, to make it clear you didn't do for yourself, and then give you a dose of any nice pleasant poison you fancy—be so good as to have it handy. I think that it might be as well for me to strangle you a bit after you were dead. Suffocation is more in the professional burglar's way than poison, and the appearances of it would prevent any of those nasty *post-mortem* examinations which the doctors are so fond of, but which must rouse feelings of indignation in the breast of any respectable corpse. I'll do it neat and comfortable for you, sir; and then I'll slip away, and nobody'll ever be a bit the wiser. Lor! what a treat it'll be for the papers—'Mysterious Death of a Gentleman!' You may comfort yourself in your last moments with the thought that your death will be a perfect gold mine to the boys who sell evening papers."

"Jebb, you are a genius!" cried the poor gentleman, looking as pleased as anything. "That's a first-rate idea. The insurance company will think me as honestly murdered as ever mortal was. Here's your money. You deserve it for inventing such a scheme, even if you weren't going to put it into execution."

Which, of course, was just what I was not going to do. I took the four five-pound notes he offered me, though I felt some scruples about it; but I dared not refuse them. I took my leave, and, I need not say, told the whole case to the nearest policeman.

The end of it was that my poor customer was certified as a lunatic, and taken to Colney Hatch. But here is a queer thing. He never had robbed the bank he was in. All the rest was true. He had gone without comforts, may be without necessaries, to give money to people who, he thought, wanted it more than he. He had insured his life for five thousand pounds, and bequeathed it to the bank; but he has never taken a penny that wasn't his. Doubtless, he had often felt tempted to do so, and the temptation, though he had resisted it, had recurred to him in his madness.

I have been down to see him at the asylum once or twice. He isn't unhappy. He seems quite contented, and the keepers say he is a help to them in looking after some of the other poor creatures who are there. I take him down some little thing from the shop when I go, and he is always very pleased to see me, and doesn't seem to remember that I was the means of his being shut up. For myself, I must say that I have a very affectionate feeling towards him; and sometimes think that I respect his madness more than some folks' sanity.

